# Impact of COVID-19 Pandemic on Trauma Theatre Efficiency

**DOI:** 10.7759/cureus.11637

**Published:** 2020-11-23

**Authors:** Nikhil Aravind Khadabadi, Peter C Logan, Charles Handford, Kishen Parekh, Munawar Shah

**Affiliations:** 1 Trauma and Orthopaedics, Walsall Manor Hospital, Walsall, GBR

**Keywords:** covid pandemic, theatre efficiency, trauma standardised operating procedures (sops)

## Abstract

Introduction

A large transformation in the management of trauma has ensued following the COVID-19 (coronavirus disease 2019) pandemic. There has been an increase in reliance on guidance for decision-making and alterations in the working of the trauma theatre. This has largely been due to the safety measures implemented. Theatre efficiency has gained increasing importance over the years, and with the added pressure of the pandemic, it is essential that trauma theatres operate efficiently. There has been no data analysing the efficiency of trauma theatre during this pandemic.

Methods and Results

We retrospectively analyzed the data at our hospital and looked into the parameters to assess trauma theatre efficiency. It was noted that the operative time and anaesthetic time went up significantly in 2020 in comparison to 2019. Also, the change over time and the late start time was significantly high in 2020. A large proportion of cases did not start on time in 2020. This resulted in a decrease in the efficiency of theatre usage.

Discussion

Reduced productivity of the trauma theatre has been due to several reasons, many of which include implementation of safety measures, such as personal protective equipment (PPE), theatre cleaning, recovery of patients, using designated routes for transfer, and many others. The challenge lies in applying these new measures into our daily practice at the same time while providing efficient care.

Conclusion

Our study highlights the key areas of concern and improvement which need to be addressed in order to render effective trauma care.

## Introduction

Trauma theatre constitutes a key part of any hospital. Its optimal utilization is vital to render effective trauma services. This is of particular importance during the current coronavirus disease 2019 (COVID-19) pandemic as it has severely affected the resources on the ground. Hence, it was prudent that we evaluate the efficiency of these services so as to advocate measures to improve its productivity.

On December 31, 2019, the first cases of pneumonia were reported. The World Health Organization (WHO) declared COVID-19 to be a global pandemic on March 11, 2020 [1-2]. This represented a major challenge to the National Health Service (NHS), and national guidelines were soon provided from NHS England with regards to changes in the clinical management of emergency and routine conditions during this crisis. This included the postponement of elective surgical services for a duration of three months with the continuation of trauma services [3]. Although trauma services continued, clinical management was adapted to ensure optimum resource utilisation. These have been supported by Standardised Operating Procedures (SOPs) from the British Orthopaedic Association (BOA) and Royal College of Surgeons (RCS) England [4-5].

The Productive Operating Theatre initiative published by NHS England on June 9, 2020 recognises that theatre teams need to work more effectively together to improve the quality of patient experience, safety, outcomes of surgical services, staff experience, and the effective use of theatre time [6]. This targeted focus not only helps theatres run more productively and efficiently but also leads to significant financial savings. In spite of all these measures, trauma theatres have been known to be inefficient and this has been widely reported in the literature [7-9]. With the added pressure of the pandemic, it is inevitable trauma theatre services will be strained.

Hence, our study was designed to look at the key parameters of theatre efficiency widely reported in the literature by Delaney et al. 2009 and Collantes et al. and evaluate its impact on the operation of the trauma theatre during the pandemic [7-8]. The data calculated included surgical time, anaesthetic time, allocated theatre time, change over time, late start time, number of daily cases, and theatre time.

## Materials and methods

Theatre efficiency during the COVID-19 pandemic was reviewed at the Walsall Manor Hospital in the West Midlands. Retrospective data from trauma theatres was obtained for April and May in both 2019 and 2020. Data was collected from a similar period during both years for comparison as the COVID-19 pandemic made its impact on the NHS during April 2020. Data was collected from the Operating Room Management Information System (ORMIS).

The theatre list ran for a full day with the same surgical, scrub, and anaesthetic team. The list began at 9 am and finished at 5.30 pm with a one-hour downtime scheduled for lunch. Weekend lists were not included as, at the time of review, they were not being implemented consistently. Only full-day theatre lists were included for comparison. The record for each operation on the database provided a summary of the procedure performed and the staff involved. The recorded parameters on the system included the time the patient entered the operating theatre and the time exiting. The start and end of anaesthetic time were recorded, along with the time of the first incision and the completion of surgery. The data was collected on an Excel® spreadsheet (Microsoft® Corp., Redmond, WA).

Based on the above data, the surgical time and anaesthetic time for each procedure, along with the change over the time between subsequent operations, were calculated. Each of these values was converted to a percentage of the total allocated theatre time. Also, the late start time was calculated, which was defined as the first patient entering the anaesthetic room more than 30 minutes after the designated time. Theatre time, which is the total duration of the time a patient spent in the theatre, was also determined.

## Results

We reviewed a total of 45 full-day lists in 2019 and 50 full-day lists in 2020 during April and May. The results from the two years are presented in Table 1. In 2019, a total of 160 procedures were conducted, whereas, in 2020, 98 procedures were carried out. The results from both the years were comparable and clearly demonstrated that a large proportion of the time was spent administering anaesthesia or performing surgery. In 2019, an average of 54% of the time was spent surgically and 17% of the time spent anaesthetically. In 2020, the percentage of surgical time and anaesthetic time dropped to 44% and 14%, respectively.

**Table 1:** Summary of Study Results

Criteria	2019	2020
Surgical time	54%	44%
Anaesthetic time	17%	14%
Allocated theatre time	7 hours, 30 minutes	7 hours, 30 minutes
Change over time	12%	19%
Late start time	17%	23%
Percentage of on-time / early start lists	35.60%	5.8%
Theatre time	113 minutes	187 minutes
Number of daily cases	4	2

**Figure 1:**
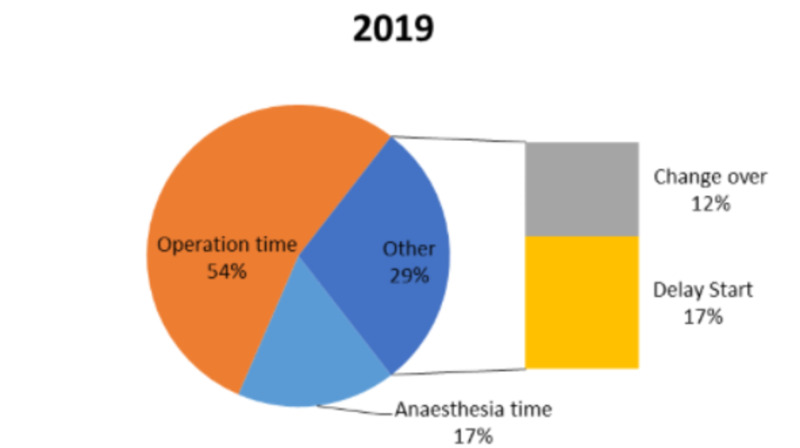
A representation of the contribution of each aspect to the trauma theatre activity in 2019

**Figure 2:**
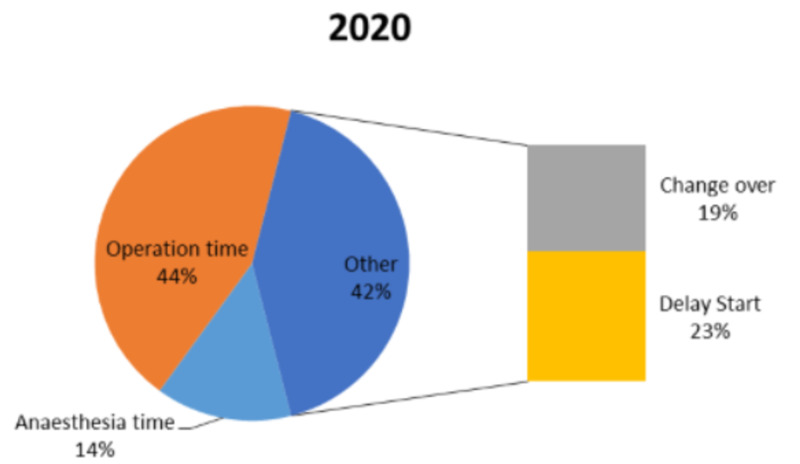
A representation of the contribution of each aspect to the trauma theatre activity in 2020

On average, in 2019, the change over time between every case was 24 minutes and this increased significantly to 38 minutes in 2020. The average daily delay start time was 16 minutes in 2019, whereas, in 2020, it was 48 minutes. The latter was surprisingly high so each start time was reviewed to assess if it was just one list that began exceptionally late which then contributed to a high average time loss. Even after excluding lists that started significantly late, the mean late start time was higher in 2020. Overall, 94.2% of lists began late in 2020 (Figures 1, 2).

**Figure 3:**
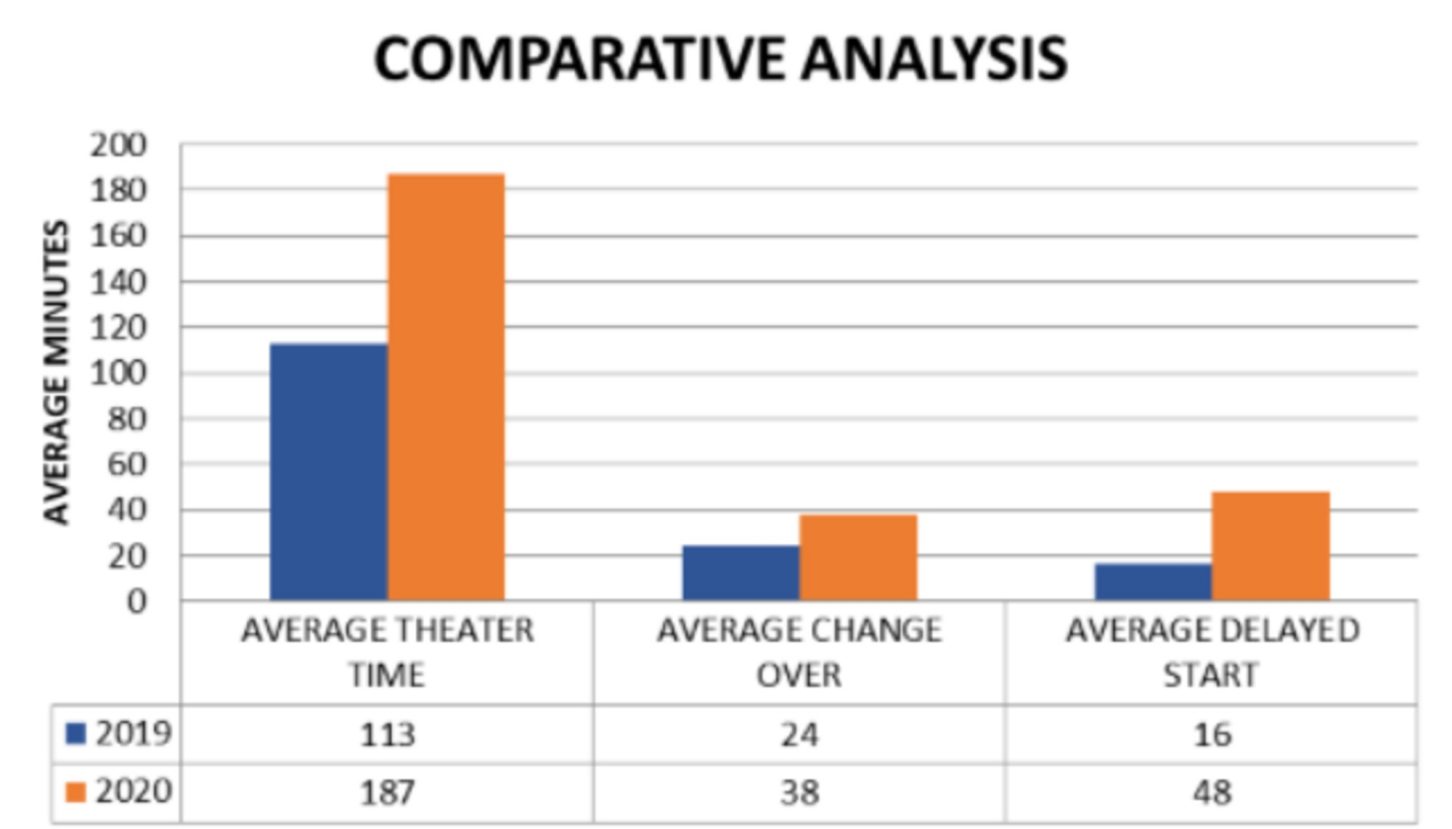
Analysis of theatre time, average change over time, and average delayed start for the years 2019 and 2020

The total mean duration of the surgical time was 73 minutes in 2019, and this increased to 90 minutes in 2020. The mean anaesthetic time was 23 minutes in 2019; this also increased to 28 minutes in 2020. The average total number of procedures being carried out in 2020 dropped significantly to two from four in 2019 (Figure 3). Also noted, the theatre time went up drastically in 2020 to 187 minutes per patient from 113 minutes in 2019.

## Discussion

Orthopaedic trauma theatres must be run in an efficient, safe, and cost-effective manner in order to remain financially sustainable and to offer maximal patient benefit [10]. COVID-19 has presented a number of particular challenges to ensuring the aforementioned necessities are maintained. In this section, the financial impact and patient impact will be discussed separately. Staff safety will also be discussed.

Within the United Kingdom (UK), healthcare is free at the point-of-care, with the cost covered by the state. Public spending is closely monitored, and budget utilisation is hotly contested. Therefore, all services must offer optimal value for the money, and the NHS is no exception. Both public and managerial expectations remain high, despite significant resource limitation. NHS services are under increasing pressure to expand and continue to provide high-quality service, whilst limiting additional expenditures [11].

Orthopaedic trauma theatres are an expensive commodity, with an estimated running cost of £24.77 per minute [10]. This does not include the additional cost of both implants and surgical devices. The results from our retrospective study have shown that, as a unit, we are completing fewer cases daily than we were prior to the COVID-19 pandemic. However, our outgoings remain high, as we are still employing the same number of staff and providing the same number of theatre sessions dedicated to orthopaedic trauma.

For the Trust itself, there is also the additional financial impact of the inevitable loss of funds. The reduced theatre case output means that patients with injuries attracting tariff incentives may wait longer for their definitive treatment, resulting in a missed tariff. This also has implications for the patient, with the potential for suboptimal outcomes and increased morbidity and mortality. It was anecdotally noted that many of our hip fractures were not meeting the best practice tariff criteria due to fewer cases being operated upon within the first 36 hours of presentation [12].

From a patient perspective, the results indicate that patients may have to wait longer for their procedure. For well patients who have suffered an injury requiring an intervention, waiting an extra few days may not lead to any adverse overall functional outcome. However, some patient’s delays can represent a very real risk to their outcome, both physically and mentally. A prolonged wait can prolong preoperative pain and discomfort, as well as increase the risk of development of other medical problems (e.g., chest infections in the immobilised patient). This may also lead to a potential delay in returning to a state of relative normality and independence, with the potential for a negative impact on the patient’s state of mind. The impact on the length of stay adds a further financial burden and ultimately negatively affects the patient experience.

Throughout the COVID-19 pandemic, the staff has continued to work in a challenging dynamic environment. However, staff safety has remained a key priority. Guidance advocated by the BOA states that the use of personal protective equipment (PPE), such as a filtering facepiece (FFP)3 mask and visor, for all surgeries using high-speed devices which are considered to be aerosol-generating [4, 13]. The American College of Surgeons recommends that surgeon(s) and personnel not needed for intubation should remain outside the operating room until anaesthesia induction and intubation are completed [14]. Firstenberg et al. advocated the opening of minimum supplies in the procedure room and runners were tasked to obtain any additional supplies for the case [15]. Postoperatively, thorough decontamination of all surfaces, screens, keyboard, cables, monitors, and anesthesia machines were advocated by Ti et al. [16]. All these measures, in turn, resulted in an increase in anaesthetic time, surgical time, change over time, and the duration of time a patient spends in the operating room in our study. This led to a fewer number of procedures being performed each day.

Over recent years, even before the COVID-19 pandemic, much research had been undertaken to investigate ways to optimise the expensive asset of trauma theatres. The concept of a golden patient is often championed as a potential solution [17-18]. The promise is that a prompt start will result in a more efficient list and is more important in these times than ever before. The concept of the golden patient is very much relevant now as it, in turn, leads to a prompt start time and increased efficiency. In our Trust, despite a golden patient being identified during the previous day, we still noticed significant delays at the start of the trauma theatre. In the initial period, this was largely due to the staff not being aware of the up-to-date protocols for patient preparation, transfer, and use of PPE.

Hospitals are ultimately a service provider, and public opinion and perception will influence their patient throughput and revenue. At the height of the COVID-19 pandemic, public support and confidence in UK hospitals were high with a nationwide appreciation for hospitals characterised by the weekly claps and frequent generous donations. As the COVID-19 pandemic continues but the case count reduces, a version of normality will return for the general public. However, adaptations to ensure safe care for both patients and staff will continue. This will result in an ongoing challenge, and we must strive to increase our efficiency to prevent delays in providing definitive care. If we fail to achieve this and patients are being routinely canceled or waiting for prolonged periods, the patient may suffer as a result. It stands to reason that were this to continue, patients and their relatives may lose confidence in the healthcare provider. Given the wide use of social media, there runs a high risk of corporate reputational damage for healthcare trusts which is difficult to rebuild once lost.

## Conclusions

The COVID-19 pandemic has had an unprecedented effect on the healthcare system in the United Kingdom. Trauma services have continued to operate during this pandemic with fewer resources due to shielding, redeployment, and sickness. Trauma theatre utilization is a key component in rendering effective trauma care. Many areas of concern noted in our study need to be considered at a wider policy level for effective delivery of orthopaedic services.
